# 

**Published:** 1921-06-11

**Authors:** 


### Royal National Orthopædic Hospital.

The Special Appeal Department of the Royal National Orthopædic Hospital is making a gallant effort to raise the £200,000 that is so urgently required for the projected building extension. Progress has been slow but sure, and all concerned are labouring with the cheery optimism which seems to be inherent in hospital workers. The fact that the grant of £24,000 from the King's Fund is conditional upon a similar amount being collected by the hospital makes it essential that no effort should be spared. The recent matinée at the Winter Garden Theatre was instrumental in raising £1,000, and the hospital benefited to the extent of over £100 from the football match played last month at the Crystal Palace between teams selected from the staffs of the L.C.C. Tramways Department and the London General Omnibus Co. The total of the subscriptions to date is about £10,000. In addition to providing increased accommodation for out-patients and for additional medical and nursing staffs, it is intended to found a School of Orthopædics, and to form a large country branch with 200 to 300 beds for children, of whom there are more than 1,000 on the waiting-list of the hospital. Included also in the project is accommodation for paying patients.

### East End Mothers' Lying-in Home.

Dr. Owen Lankester, presiding at the annual meeting of the East End Mothers' Lying-in Home, said that the Home has during the past twelve months continued to flourish. It has received the commendation of the Ministry of Health, and that Department has accorded them financial help. Steps have been taken to remedy the somewhat overcrowded condition of the wards, and if they can afford it the property next to the Home will be acquired as an extension of the existing premises. Miss Lena Ashwell made an eloquent appeal for increased financial support for the Home, which, she said, is one of the most delightful hospitals she has ever visited. A better, kinder, cleaner, and happier place it would not be possible to find. She considers it a privilege to help such an institution, and expressed the opinion that people who take a real, as opposed to a casual, interest in their poor East End sisters will get more happiness out of life. The report states that 988 in-patients were delivered, 972 infants were born alive, 34 were stillborn, and there were 18 twins. Out-patients delivered numbered 1,481, and 1,479 infants were born alive. District visits paid totalled 28,635. The total number attended during the year was 2,469. There were no deaths in either department. The total income for the year amounted to £6,129, and exceeded the expenditure by £356. Several babies from the Home, to whom Dr. Owen Lankester humorously referred as "specimens," were present.

### The Great Fair for King's College Hospital.

It is greatly to be hoped that the weather will be propitious to the Great Fair which is being held from June 9 to June 21 in the grounds of "The Falcons," kindly lent by Lieut.-Colonel Sir R. A. Sanders, Bart., M.P., in aid of King's College Hospital, daily from 12 noon to 10.30 p.m. Here Londoners will be able to revel in a real country fair, where every attraction of the showman's art will be provided by the British Showmen's Guild. Admission after the opening ceremony is only 6d. for adults and 3d. for children, and we hope that parties will be made up and that schools will send their scholars to participate in all the glories which every child—and we are all children at a Fair—so radiantly enjoys. And whilst we are enjoying ourselves we shall be assisting King's College Hospital and helping to open its closed wards.

### New Scheme at Cardiff.

In consequence of the financial difficulty of King Edward VII. Hospital, Cardiff, the Finance Committee recommends that all patients who contribute to organisations at a certain weekly rate should be exempt from personal payments to the hospital; that all others shall contribute according to their means; that on receiving a ticket of application for admission the hospital shall inquire of the patient by letter or interview what he can pay; that the sum agreed shall be entered on the patient's record papers and collected as a matter of routine on his admission. Tickets should contain printed questions asking where and how the recipient has contributed to the hospital. Lastly, no precedence should be given to any patient because he has paid.

When these recommendations had been submitted, Captain T. O. Edwards said that the hospital was about £70,000 in debt, and was spending each week £1,000 more than its income. He believed the costs per patient were very high, and that the officials were almost as numerous as the patients. Mr. Leonard Rea replied that the situation was too serious to last. It was because about 40 per cent. of the patients did not contribute to the hospital in any way that the present scheme was proposed. Sir William Diamond, Chairman of the Board, deprecated distinctive criticism, and the recommendations were unanimously agreed to.

### Presentation to a Doctor.

Dr. G. Fuller England has now been presented with a silver tray on the occasion mentioned in the inscription, which runs: "Presented to G. Fuller England, Esq., M.D., by his Colleagues and the Committee of the Royal Hants County Hospital on his retirement as honorary physician, in appreciation of the good services rendered, and of the many years of pleasant association in carrying on the work of the hospital. January 20, 1897—February 9, 1921."

### Carnegie Trust Health Schemes.

The seventh annual report of the Carnegie United Kingdom Trust refers to its various activities in the domain of health work. At the six model Child Welfare Centres for which grants have been promised the Motherwell Centre is the only one actually in course of erection. In three other cases—Shoreditch, Birmingham, Liverpool—plans have been passed, and it is hoped that building will soon be possible. In the case of Rhondda the difficulty of obtaining a site has been an obstacle up to the present. Dublin Corporation has unfortunately been unable, owing to obvious causes, to take any steps. The total of the grants promised for these six Centres is £130,000. In regard to hostels, the trustees proposed to limit themselves to the provision of a single hostel, in Stornoway, but the lowest tender submitted was more than 50 per cent. above the maximum allotted. New plans on a more modest scale have been approved. A grant of £1,000 annually for five years to the National Institute of Industrial Psychology is of particular interest, since it is an attempt to assist in its pioneer stages a voluntary body of persons interested in the health side of industrialism. The Institute is complementary to the official body, the Industrial Fatigue Research Board, and proposes to undertake certain inquiries which that body is not under instruction to conduct. It will also advise manufacturers as to factory conditions, economy of labour, and so forth.

### PASSING EVENTS AND TOPICS.

### Alexandra Day, June 22.

GREAT efforts are being made to ensure that the success of the coming Alexandra Day shall be commensurate with the great needs of the hospitals. Since the inauguration of the "Day," ten years ago nearly a million pounds has been collected. All those who will assist the organisers of the "Day" by selling roses or in other ways are asked to communicate at once with Miss May Beeman, 33 The Grove, The Boltons, S.W.

### University College Hospital Ladies' Association.

LIKE most other London hospitals, University College Hospital is in financial difficulties. In order to help, the Ladies' Association held a Sale on Wednesday, June 8, Major Harold and Lady Zia Wernher kindly lending their London residence (Someries House, Regent's Park) for the purpose. Her Highness Princess Helena Victoria opened the Sale. The things offered for sale were of a varied nature, including fruit and other farm and garden produce. There were also some special features, such as an Antique Stall (arranged by Mrs. Arthur Platt) and a Second-hand Book Stall. The splendid work done for University College Hospital by its Ladies' Association, whose latest development is the establishment of the Infant Welfare Department, should ensure a splendid financial result from this Sale.

### Pound Day at the Royal Westminster.

THE matron of the Royal Westminster Ophthalmic Hospital, King William Street, West Strand, will be grateful for gifts in money or in kind on the occasion of the Pound Day which will be held at the hospital on Tuesday, June 21. The hospital will be open to the public from three till seven, and tea will be served from four to five. The nurses will have a stall for the sale of fancy articles.

### Medical and Other Appointments.

Mr. H. Middleton, M.B., Military Cross, assistant medical officer of health for Denbighshire, has been appointed medical officer of health and schools medical officer for Pembrokeshire.

Mr. A. G. Timbrell Fisher, M.C., F.R.C.S., has been elected surgeon, with charge of out-patients, at the Dreadnought Hospital, Greenwich, in succession to Mr. E. T. C. Milligan, O.B.E., F.R.C.S., who has been promoted to the senior staff.

Dr. J. H. Douglas Webster has been appointed honorary physician-in-charge of the department of physical medicine at the Middlesex Hospital. Dr. Webster, who is honorary radiologist to the Manchester Ear Hospital and radiologist to the Manchester Board of Guardians, is an Edinburgh graduate and obtained his M.D. in 1907.

The Rev. F. H. Bettison has been appointed Secretary-Superintendent of the Convalescent Home, Redcar. The Governors desire to thank all who applied for the appointment.

### Ancient Hospitals.

"Great Thoughts" for June contains a thoughtful note on "Hospitals and Christianity" by Miss C. U. Kerr, LL.A., a contributor to these pages, who is the Welfare Supervisor at the Co-operative Wholesale Society's Stores at Irlam, Manchester.

### The Prince of Wales and the Red Cross.

The Prince of Wales has sent a message to the League of Red Cross Societies that the effort which the League of Red Cross Societies is making to serve humanity in peace time as well as in war has his heartiest approval, and he trusts that the campaign now being undertaken will result in a very large addition to the membership of National Red Cross Societies. The British Red Cross Society hopes that volunteers will offer their services to help the suffering in peace time in the same spontaneous manner as they came to the aid of the sick and wounded in war time. The Red Cross should be available on all occasions when the clergy and heads of educational establishments, such as orphanages, require the simple assistance which is often lacking on such occasions as school and choir treats and annual outings.

### The General Nursing Council for Scotland.

At a meeting of this Council, held at 13 Melville Street, Edinburgh, on Wednesday, June 1, the Registrar submitted correspondence passing between him and the Registrar of the English Council. This was considered, and the Registrar was instructed to point out to the English Council that the Scottish Council are as strongly opposed as the English Council to the proposal of the Scottish Board of Health to certify for registration in the general part of the Register existing nurses on the Board's Fever Register, and that the delay in having the Scottish Council's Rules approved by the Board is due to their objections to this proposal.

Authority was given to the Syllabus Committee to prepare a short examination syllabus suitable for distribution to intending probationers.

### The Irish Register.

### NUMEROUS APPLICATIONS.

A meeting of the Registration Committee of the General Nursing Council for Ireland was held on Thursday, June 2, at the offices of the Irish Public Health Council, 33 St. Stephen's Green, Dublin, to deal with the numerous applications received for admission to the Irish Register of Nurses. Sir Edward Coey Bigger presided, and there were also present: Miss M. Huxley (matron, Elpis Private Hospital, Dublin, Vice-Chairman), Colonel Sir William Taylor, C.B., F.R.C.S.I., Miss Reeves (matron, Dr. Steeven's Hospital), Miss O'Flynn (matron, Children's Hospital, Temple Street, Dublin), Miss Michie (superintendent, Queen Victoria Jubilee Institute for Nurses), and Miss Matheson (Hon. Secretary, Irish Board, College of Nursing).

The Committee hope to meet every three weeks to deal with the applications from existing nurses, as these cases must be dealt with within the statutory period of grace if they are to obtain the benefit of admission without examination.

### Letters to a Nurse.*

The reader will find in this little volume some very pleasant correspondence from a wise and humorous uncle to a wayward modern niece, who has chosen the thorny path of a general nursing training. He understands her difficulties, explains her lectures (we cannot help thinking she was badly taught in her hospital), and, more than anything else, Uncle Luke teaches Barbara to look at life from the point of view of other people; to understand the harassed sister, the overtired senior nurse, and to see the dumb appeal in a nervous patient's eyes. She will be a good nurse if she heeds all his teaching, and so will many another who may do the same. He deals harshly with the present system of training for nurses, but his frank criticism is not unjust. No hospital can afford to say, "Lord, I am not as other men." "Letters to a Nurse" is a little book worth reading.

* *Letters to a Nurse.* By a Midland Doctor. (John Bale, Sons, and Danielsson, Ltd. Price 5s. net.)

June 11, 1921] [Price Twopence

### The Hospital

**RATES OF SUBSCRIPTION** (post free, payable in advance).

Three months, 3/3; six months, 6/6; twelve months, 13/-. (Should the cost of printing and paper as well as increased postage, necessitate alteration of these rates, the period paid for will be reduced proportionately on unexpired subscriptions.) THE HOSPITAL "Index" to Volume LXIX., October 1920 to March 1921, is now ready, price 1/1 per copy, post free. Address The Manager, THE HOSPITAL, "The Hospital" Building, 28/29 Southampton Street, Strand, London, W.C. 2.

